# The temporal trend of women’s cancer in Changle, China and a migrant epidemiological study

**DOI:** 10.3389/fonc.2023.1092602

**Published:** 2023-03-16

**Authors:** Yu Chen, Mengjie Song, Yanyu Zhang, Xingxing Yu, Shuqing Zou, Pingxiu Zhu, Yulin Zhou, Haomin Yang

**Affiliations:** ^1^ Department of Epidemiology and Health Statistics, School of Public Health, Fujian Medical University, Fuzhou, China; ^2^ United Diagnostic and Research Center for Clinical Genetics, Women and Children’s Hospital, School of Medicine and School of Public Health, Xiamen University, Xiamen, Fujian, China; ^3^ Department of Medical Epidemiology and Biostatistics, Karolinska Institutet, Stockholm, Sweden

**Keywords:** women’s cancer, incidence, mortality, joinpoint analysis, Chinese immigrant

## Abstract

**Background:**

Although the etiology of women’s cancer has been extensively studied in the last few decades, there is still little evidence comparing the temporal pattern of these cancers among different populations.

**Methods:**

Cancer incidence and mortality data from 1988 to 2015 were extracted from the Changle Cancer Register in China, and cancer incidence data for Los Angeles were extracted from Cancer Incidence in Five Continents plus database. A Joinpoint regression model was used to analyze the temporal trends of incidence and mortality for breast, cervical, corpus uteri and ovarian cancers. The standardized incidence ratios were applied to compare the cancer risk across populations.

**Results:**

An increasing trend of incidence rate for breast, cervical, corpus uteri and ovarian cancer was observed in Changle, although the rate leveled off for breast and cervical cancer after 2010, although not statistically significant. The mortality rate of breast and ovarian cancer was slightly increased during this period, while we found a decreased mortality of cervical cancer from 2010. The mortality of corpus uteri cancer showed a decreasing and then increasing trend. The incidence of breast, corpus uteri and ovarian cancer in Chinese American immigrants in Los Angeles was significantly higher than indigenous Changle Chinese and lower than Los Angeles whites. However, the incidence of cervical cancer in Chinese American immigrants shifted from significantly exceeding to lower than Changle Chinese.

**Conclusion:**

The incidence and mortality of women’s cancers in Changle were generally on the rise, and this study concluded that environmental changes were important factors affecting the occurrence of these cancers. Appropriate preventive measures should be taken to control the occurrence of women’s cancers by addressing different influencing factors.

## Introduction

Women’s cancers involve cancers of the breast, ovaries, uterus, cervix, vagina, and vulva. Breast cancer is the most common cancer among women world-wide, followed by cervical cancer, while ovarian cancer is the deadliest gynecological malignancy worldwide (1, 2). In high-income countries, incidences of the breast, cervical and ovarian cancers are all on a downward trend, while the incidence of corpus uteri cancer has increased from 1976 to 2012 (3). In China, the medical expenses of the three gynecological cancers increased instead of decreasing, which caused a heavy economic burden (4).

In the past few decades, the etiology of women’s cancers has been researched extensively. For example, a large number of epidemiological studies have manifested that socioeconomic status has a considerable impact on the occurrence and development of these cancers (5, 6). A subtle difference in certain genes can explain a part of the observed disparities in breast and gynecological cancers between various populations in numerous genetic studies (7, 8). The true etiology of women’s cancers is difficult to understand, given that it has become increasingly clear that cancer development cannot be viewed as purely genetic or purely environmental.

Studies of migrants are classic tools for exploring the significance of environmental, social, and genetic traits in disease etiology and have been particularly important for disentangling the etiology of cancer (9). Earlier studies, which initially focused on individuals in one region, found that the incidence of women’s cancers varies significantly between different ethnic groups (10–12). Studies of migrants from China, Japan and the Philippines to the USA have shown that increased rates of breast cancer are first observed after 10 years of residence in the USA, whilst the maximum increase is not seen until the offspring of migrants have been resident for a generation or two (13). However, evidence for gynecological cancers in Chinese immigrants is scarce.

Chinese immigrants are the largest Asian ethnic group in the USA, accounting for about 36.2% of immigrants in California, and they are among the groups with the oldest immigration history (14). Meanwhile, Changle is a district in southeast China with a long history of immigration, and most of the immigrants went to the USA. In 2003, there were 170,000 immigrants from Changle in the USA together with their families, which is one fifth of the original population (15). Therefore, Chinese women in Changle could be considered an appropriate reference for migrant study of women’s cancers. At present, breast cancer is one of the leading diseases that threatens the health of women, and cervical and ovarian cancers also ranked among top ten causes of cancer death in women in Changle (16).

However, previous study showing the rising trend of women’s cancer in Changle was updated until 2002 (17), and it is still unknown about the temporal trend of women’s cancer risk in recent years, when China has experienced rapid economic growth. Moreover, the few studies on the differences in risk of women’s cancer between Chinese and other ethnicity mainly focused on breast cancer (18, 19), while the only migrant study comparing breast cancer risk among indigenous Chinese, Chinese Americans and White Americans was conducted during 1968-1981 (20). Considering the changing social and environmental factors in China after the reform and opening up, it is necessary to investigate the temporal risk pattern of women’s cancer in indigenous Chinese in the recent 30 years, and compare it with other populations.

In the current study, we compared the incidence rates and standardized incidence ratios of breast and gynecologic cancers among indigenous Chinese in Changle, Chinese Americans and Los Angeles whites. The temporal trends in women’s cancers were investigated in Changle Chinese population. Findings in this study can not only provide the fundamental information for cancer prevention and control, but also explore the etiology by comparing three populations.

## Materials and methods

### Study data

The data for women’s cancer in Changle District were extracted from the Changle Cancer Register in southeast China, from 1988-2015. The Changle Cancer Registry was established in 1986, with the support from Changle Institute for Cancer Research, Fujian Medical University and The University of Hong Kong. All medical facilities that diagnose and treat cancer in the region are required to report all newly diagnosed cancers and deaths resulting from cancer, using a standardized notification card and medical certification of death. The demographic information was registered by calendar year, sex and 5-year age group. The quality of the registration was relatively good (21), and it had reported data to National Central Cancer Registry of China and Cancer Incidence in Five Continents from 1988.

Cancer incidence for Chinese Americans and white Americans who were residents of Los Angeles, the USA were extracted from Cancer Incidence in Five Continent Vol. Plus (CI5plus, http://ci5.iarc.fr/CI5plus) from 1988 to 2012. Since 1972, the Cancer Surveillance Program has routinely collected and analyzed information on all cancer diagnoses made among residents of the county. The original cancer data is categorized by gender, age and cancer sites, which are identified *via* the International Classifications of Diseases, 10^th^ version (ICD-10).

With the available cancer data in Changle, Chinese Americans in Log Angles and Los Angles whites, the study analyzed age-standardized incidence and age-standardized mortality of women’s cancer, including breast [C50], cervical uteri [C53], corpus uteri [C54], and ovarian [C56] cancers.

### Patient and public involvement

Patients or the public were not involved in the design, or conduct, or reporting, or dissemination plans of our research.

### Statistical analysis

The segi’s standard world population was used to estimate the age standardized rates of women’s cancers per 100,000 person years, for all groups. The annual percentage change (APC) and average annual percent change (AAPC) were used to quantify the trends for age standardized incidence and age standardized mortality. A regression model was calculated by Joinpoint Regression Program 4.3.1.0 which was developed by the Statistical Research and Applications Branch, National Cancer Institute (https://surveillance.cancer.gov/joinpoint/). The model fitted the natural logarithm of the age-standardized incidence and age-standardized mortality. The independent variable is the calendar year. Analysis starts with the minimum number of joinpoints (*i.e.*, 0 joinpoint, representing a straight line) and tests for model fit with a maximum of 2 or 3 joinpoints. R^2^ was calculated to choose the best fit model for the joinpoint regression. We also calculated AAPC for each 5-year interval period as a sensitivity analysis.

The standardized incidence ratio (SIR), a ratio of observed to expected number of cases, was used to compare the incidence of women’s cancers among the three populations, using Chinese Americans as the reference population. The expected number of cases was calculated by multiplying the number of population in age (5-year categories) and calendar-specific strata of the Changle Chinese and Los Angles white women by the incidence rate of each outcome in the corresponding strata of Chinese Americans. The ninety-five percent confidence interval (CI) of the SIR was calculated based on the Poisson distribution method described by Vandenbroucke (22), and potential heterogeneity of SIRs by populations was examined using Chi-Square tests. A p value <0.05 was considered statistically significant.

## Results

### The temporal trend of women’s cancer in Changle district

**Tables 1**, **2** show the crude incidence rate, the crude mortality rate, age standardized incidence rate and age standardized mortality rate by cancer site at the beginning (1988) and end (2015) of the study period, together with results of Joinpoint analysis for age standardized incidence and mortality in women, respectively.

**Table 1:** Crude incidence rates, age standardized incidence and Joinpoint analyses for 1988 through 2015 in Changle.

Site	Year1988-1992		Year 2015		Joinpoint analysis (1988-2015)
	Crude rate (/100 000)	Std. Rate (/100 000)	Crude rate (/100 000)	Std. rate (/100 000)	AAPC(95%CI)
Breast	4.60	5.40	20.21	13.93	3.3*(-4.7,12.0)
Cervix	0.90	1.20	12.78	8.59	10.7**(7.5,14.1)
Corpus uteri	2.10	2.50	5.35	3.57	5.1**(1.8,8.6)
Ovary	0.90	0.80	3.86	3.24	5.1**(3.0,7.2)

AAPC, average annual percent change (%); CI, confidence interval; Std. rate, age standardized rate.

*The average annual percent change is significantly different from 0 (two-side p<0.1).

**The average annual percent change is significantly different from 0 (two-side p < 0.05).

**Table 2:** Crude mortality rates, age standardized mortality and Joinpoint analyses for 1988 through 2015 in Changle.

Site	Year1988-1992		Year 2015		Joinpoint analysis (1988-2015)
	Crude rate (/100 000)	Std. Rate (/100 000)	Crude rate (/100 000)	Std. Rate (/100 000)	AAPC
Breast	2.80	3.40	5.35	3.55	0.6(-0.6,1.9)
Cervix	0.50	0.70	2.68	1.92	5.1(-1.9,12.7)
Corpus uteri	1.90	2.40	3.57	2.34	0.7(-15.7,20.3)
Ovary	0.50	0.40	1.78	1.28	3.7(-4.8,12.9)

AAPC, average annual percent change (%); CI, confidence interval; Std. rate, age standardized rate.

**Figure 1:**
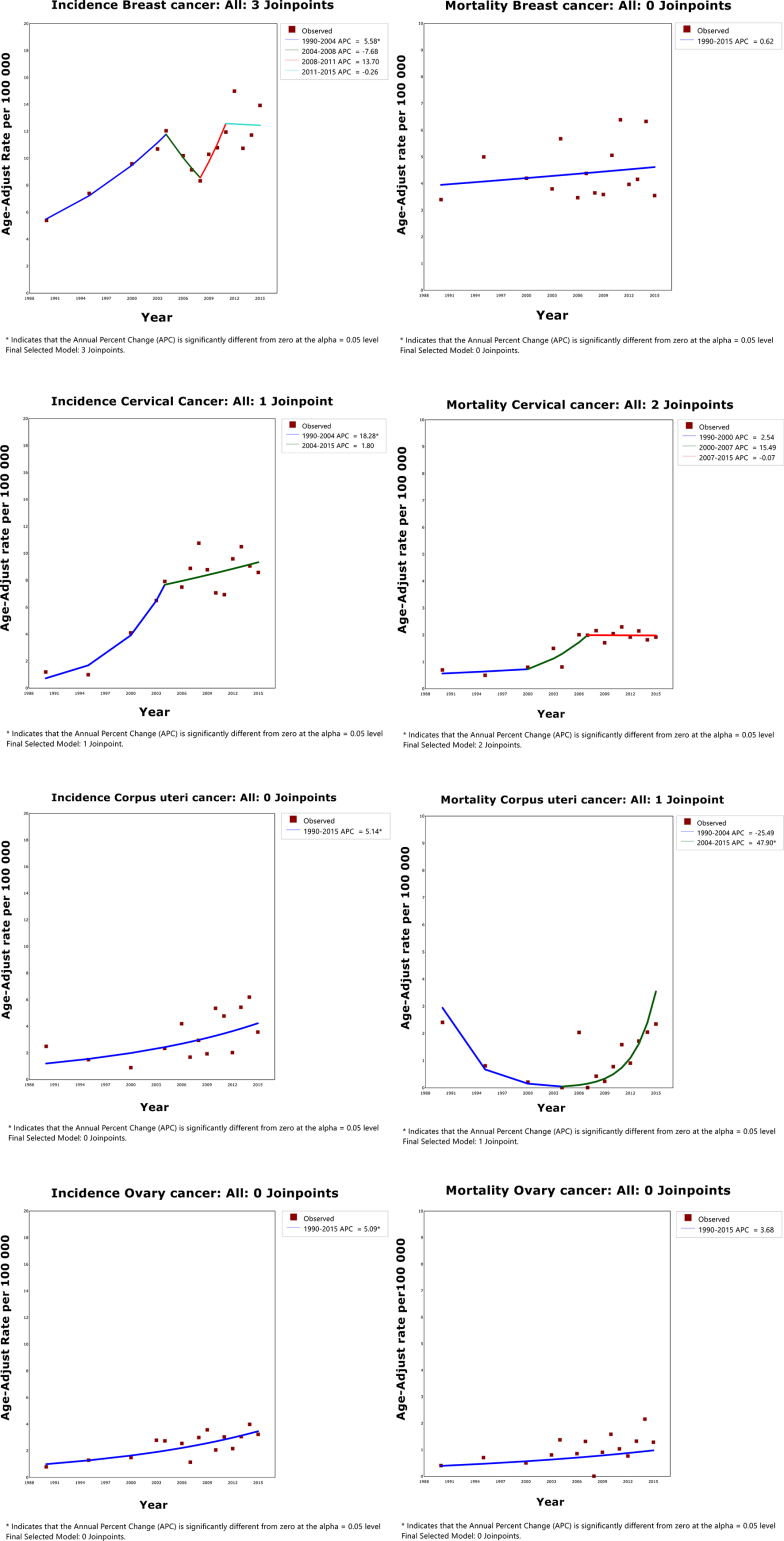
Trends in cancer incidence and mortality in Changle.

Overall, for people in Changle, the age-standardized incidence of breast, cervical, corpus uteri and ovarian cancers markedly increased during the study period (**Figure 1** and **Supplementary Table 1**), with the most obvious increase observed in cervical cancer (AAPC=10.7, 95%CI=7.5-14.1). For breast cancer, the trend fluctuated, increasing by 5.6% per year from 1988 to 2004, declining by 7.7% per year from 2004 to 2008, rising by 13.7% per year from 2008 to 2011, and stabilizing from 2011 to 2015. The trend of cervical cancer was divided into two segments, clear consecutive increasing by 18.3% from 1988 to 2005, and slowly rising by 1.8% from 2005 to 2015. Whereas, the age standardized incidence for cancers of the corpus uteri and ovary continued an upward trend from 1988 to 2015.

From 1988 to 2015, there was no significant change in the standardized mortality rates for women's cancer (**Supplementary Table 2**). In cervical cancer, the trend is divided into three parts, with age standardized mortality increasing during the study period. On the contrary, the tendency of the age-standardized mortality of corpus uteri cancer showed a U-shaped curve, decreasing by 25.5% per year from 1988 to 2005, and rising by 47.9% per year from 2005 to 2015.

As sensitivity analyses, when the maximum number of joinpoints varied between 2 and 3, majority of the results did not change. However, the number of connections for the standardized incidence rate of breast cancer (maximum of joinpoint=2, R^2 =^ 0.70; maximum of joinpoint=3, R^2 =^ 0.92) and the standardized mortality rate of cervical cancer (maximum of joinpoint=2, R^2 =^ 0.86; maximum of joinpoint=3, R^2 =^ 0.74) differed a lot. Therefore, to make a better fit for the data, the maximum of joinpoint for the standardized incidence rate was set to 3 and the mortality rate was set to 2. In addition, AAPCs of the four cancers for each 5-year interval period were also presented in **Supplementary Table 3**.

### The disparities in women’s cancer among different populations

**Figure 2:**
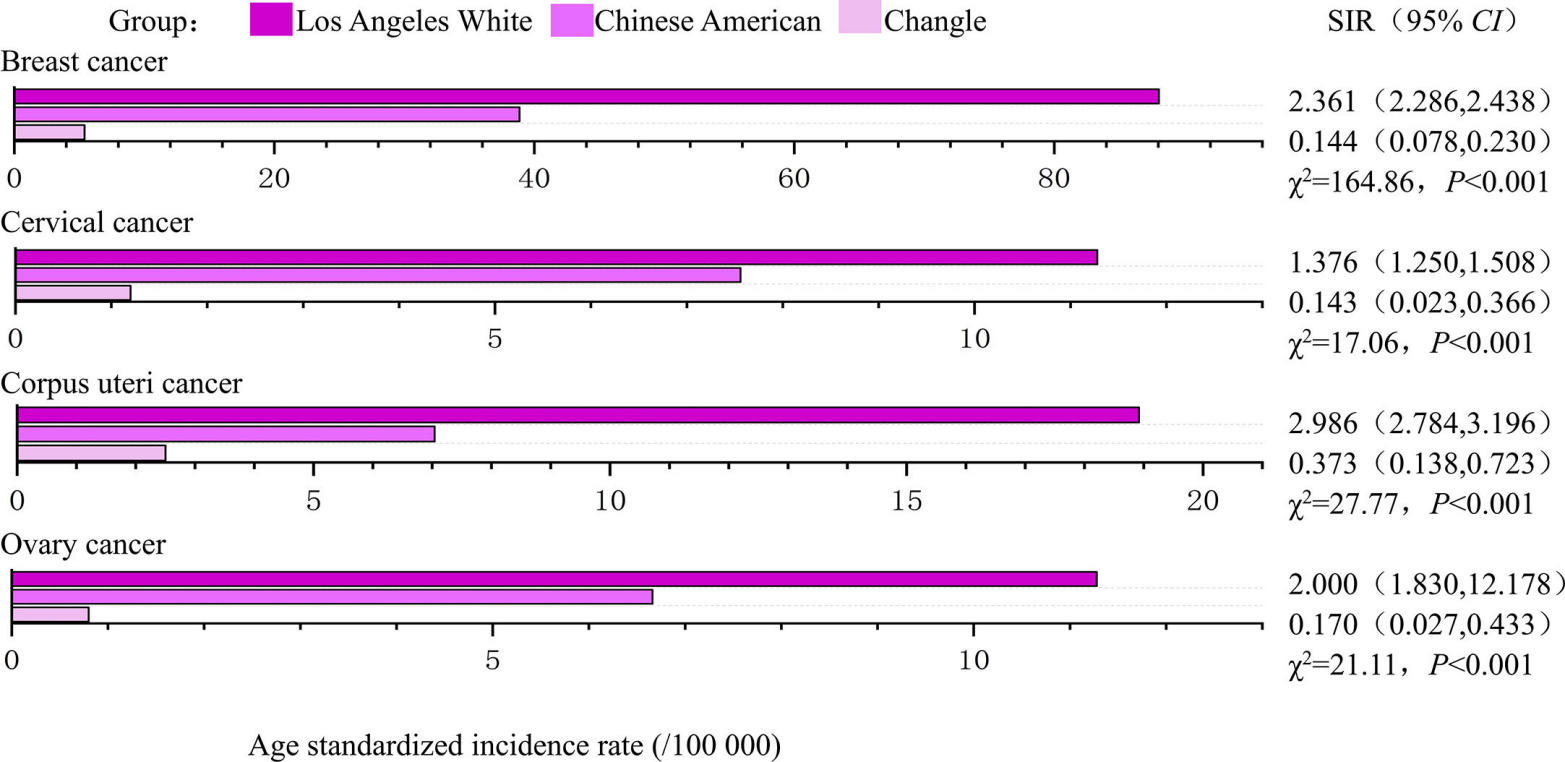
The age-standardized incidences and disparities in women’s cancer in 1988-1992. SIR, standardized incidence rate.

**Figure 3:**
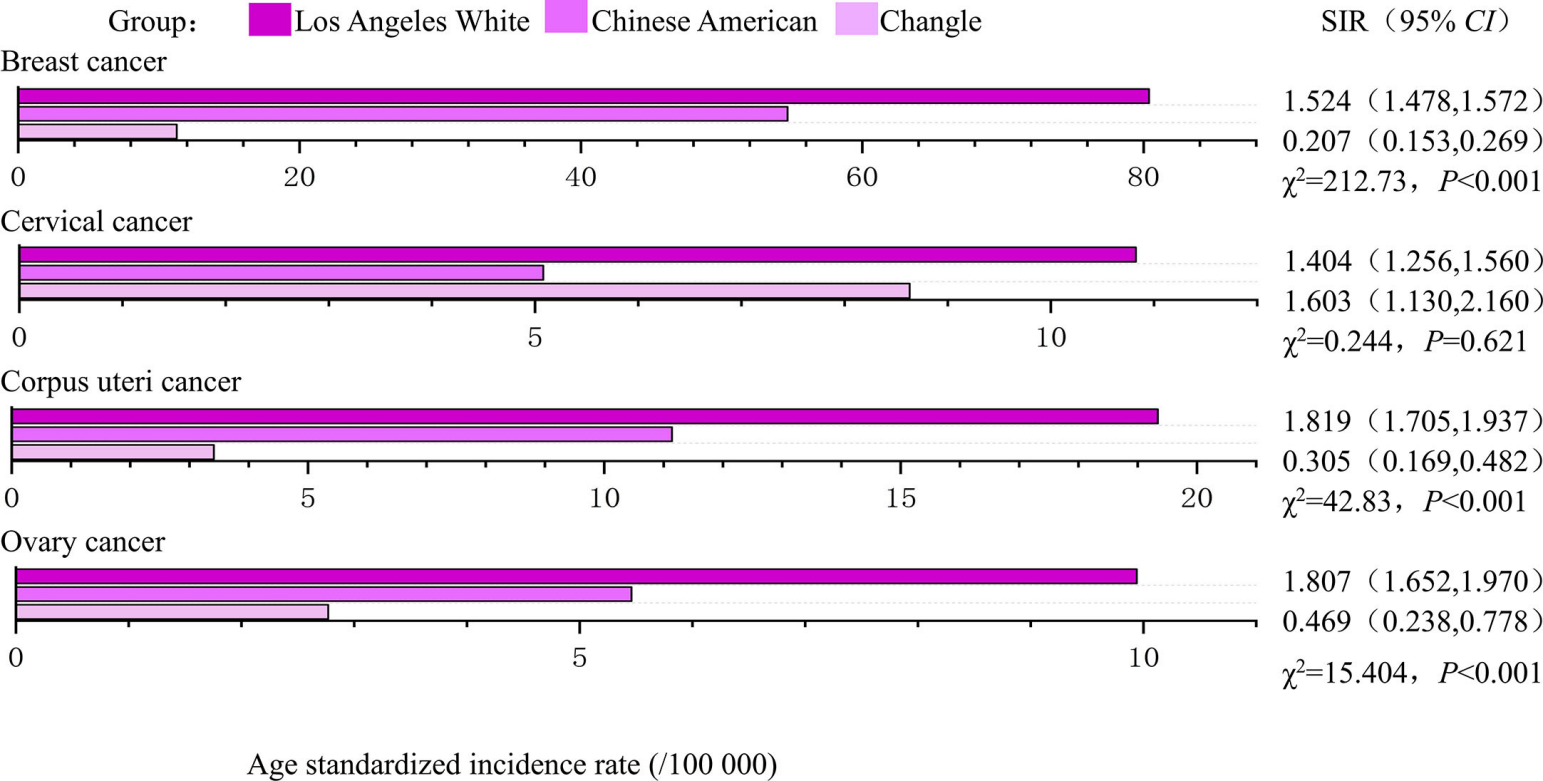
The age-standardized incidences and disparities in women’s cancer in 2008-2012. SIR, standardized incidence rate.

During the study period (1988-1992 and 2008-2012), the age standardized incidence of breast, corpus uteri and ovarian cancers were highest in Los Angeles White, intermediate in Chinese Americans and lowest in indigenous Changle Chinese populations, regardless of years (**Figures 2**, **3**). For example, Los Angeles whites had a significantly 2 times higher risk of breast cancer from 1988 to 1992 when compared with the Chinese American population (SIR=2.361, 95% CI 2.286-2.438), whereas women in Changle had a significantly lower risk of developing breast cancer, (SIR =0.144, 95% CI 0.078-0.230). In 2008-2012, the effect size was slightly attenuated with a1.5-fold increased risk in Los Angeles whites (SIR=1.524, 95% CI 1.478-1.572), and an 80% reduced risk in Changle (SIR=0.207, 95% CI 0.153-0.269). For ovarian cancer, the disparities in age standardized incidence were also statistically significant among the three populations. These results suggested that over the past 20 years, the incidence of breast and ovarian cancer among these three populations has trending closer to each other. For corpus uteri cancer, the incidence rate in Los Angeles whites was also close to that in Chinese Americans, but the gap between the incidence rate in Changle residents and Chinese Americans has slightly widened. However, the difference is not statistically significant.

Los Angeles Whites had a nearly 40% increased risk of cervical cancer, compared to that in Chinese Americans in both time periods (1988-1992: SIR= 1.376, 95% CI 1.250-1.508; 2008-2012: SIR= 1.404, 95% CI 1.256-1.560). In 1988-1992, even more striking discrepancies were detected between Changle residents and their counterparts among Chinese Americans in Los Angeles (SIR= 0.143, 95% CI 0.023-0.366). However, in 2008-2012, the age-standardized incidence of Changle residents had obviously risen for cervical cancer, with a risk increase of 1.6-fold (SIR= 1.603, 95% CI 0.130-2.160), compared to the Chinese American population.

## Discussion

### Key results

Generally speaking, age standardized incidence and mortality for women’s cancer in Changle have been rising for the majority of cancers. The few exceptions showed an increase after decrease in mortality of corpus uteri cancer. For breast, corpus uteri and ovarian cancers, we found a higher incidence in Los Angeles Whites than in Chinese Americans, and lower incidence in Changle residents than in Chinese Americans. We also observed a changing difference between cervical cancer incidence in Chinese Americans and the Changle Chinese. Overall the incidence of women’s cancers among Chinese-Americans is closer to that of Los Angeles whites. This suggests that changes in lifestyle and living environment are associated with increased incidence of breast, cervical,corpus uteri and ovarian cancers.

In general, for women’s cancers, the risk in migrants from China has reached a medium level between the country of origin and the country of new residence. Similar patterns of change have been reported in studies previously limited to other Asian-American immigrants, such as immigrants from Japan (23), South Korea (24) and the Philippines (25). In our study, we also showed that Los Angeles white residents had a significantly higher risk of women’s cancer than Chinese Americans and Changle Chinese. This result is supported by other studies showing that cancers of the breast, corpus uteri, and ovary are more common in high-income countries than in middle- or low-income countries (26–28).While these disparities have persisted over period, the gap between the three groups has narrowed to varying degrees, suggesting a changing of associated social and environmental factors.

In the current study, we found an increased incidence and mortality of breast and ovarian cancer in Changle, which was similar to the trend in other rural areas of China (29, 30). In addition, the incidence of breast and ovarian cancer increased after Chinese women migrated to the USA and was closer to that of white Los Angeles. Although breast and ovarian cancer have a heritability of 30-40% and BRCA1/2 mutations are well known for both cancers (31), these results in our study suggested more pronounced effect from social and environmental risk factors for breast and ovarian cancers, while genetic factors may have a smaller contribution to breast and ovarian cancer in Chinese women (32).

The incidence of corpus uteri cancer increased each year during the study period. Notably, its mortality rate showed a decreasing trend from 1988-2005, and an increasing trend from 2005-2015, but not statistically significant. Improvements in treatment techniques and increased incidence might contribute to this trend in mortality. Considering the increasing incidence of corpus uteri cancer in Changle and Chinese Americans approaching to the incidence of Los Angeles whites, it is reasonable to believe that patterns of corpus uteri cancer may change as the environment changes.

For cervical cancer, the incidence and mortality rates increased by an average of 10.7% and 5.1% per year, respectively. The magnitude of its change is greater than the average level in China (33). This may be explained by increased HPV exposure, inadequately compensated for by the benefit from cytological screening (12). Notably, the incidence rate of cervical cancer among Changle residents was significantly lower than that of Chinese Americans from 1988 to 1992, but gradually exceeded that of Chinese Americans from 2008 to 2012. This changing trend might not only reflect the prevention and control efforts for cervical cancer, but also suggest the present and future public health concern in terms of this cancer in Changle district. A review of the relevant literature revealed that routine cervical cancer screening by cervical cytology was introduced in the United States in 1950 (34) and California launched a universal free cervical cancer screening program and treatment for precancerous lesions in 2002 (35). As of 2010, the rates of cervical cancer screening completed in the past three years among whites in the United States and American Chinese were 83.4% and 71.6% respectively (36). HPV vaccination has been a priority for cervical cancer prevention in the United States since 2008 (37). However, The free cervical cancer screening program in Changle district only started in 2012 and has limited coverage of the population (38). The screening rate of cervical cancer in China was only 26.7% in 2013 (39). Therefore, the cervical cancer screening process might play a role in the cancer incidence trend.

Some limitations of our study should be mentioned here. First, improvements in medical treatment and cancer registration system, and the increase in cancer diagnosis rate and reporting rate might have affected the long-term trend analysis. Second, we don’t have individual level information on the study participants, which means it is impossible to accurately link shifts in cancer patterns to specific environmental changes. Third, there is no information on the generational (e.g., first, second, or third generation) or immigration status of Chinese in Los Angeles, which may pose a comparability problem to some extent. Fourth, time of migration was not available in the dataset and we did not take into account the “healthy migration effect”, in which new immigrant populations are healthier than native populations in their countries of origin and places of migration, which may introduce bias when comparing cancer incidence rates between immigrant populations and their native populations and populations in places of migration. However, the observed higher risk of cancer in those women who migrated compared to the native population argued against the potential healthy migration effect between them. Considering these limitations, our results should be interpreted with caution.

## Conclusions

In summary, in the current study, we analyzed the trend of women’s cancer incidence in the Changle district, and compared the incidences of these cancers among Chinese Americans, Los Angeles whites and Chinese populations in the Changle district. These results suggest that the risk of women’s cancers in Changle is increasing year by year and environmental factors might have an indelible impact on these cancers, thereby providing new insights into cancer genesis and prevention.

## Data availability statement

The raw data supporting the conclusions of this article will be made available by the authors, without undue reservation.

## Ethics statement

The study was based on publicly available data of population sizes and aggregated number of cancer cases. Therefore, ethics approval or consent to participate was not necessary.

## Author contributions

YC and MS had full access to all data, and take responsibility for the integrity of the data and the accuracy of the analysis. Study concept and design: HY and YZhou. Acquisition, analysis, and interpretation of data: YZhag, MS, HY, and YC. Drafting of the manuscript: YC and YZhou. Critical revision of the manuscript for important intellectual content: All authors. Statistical analysis: YC and MS. Funding: HY. All authors contributed to the article and approved the submitted version.
