# Supplementary material for: The temporal trend of women’s cancer in Changle, China and a migrant epidemiological study
Source: Front Oncol. 2023 Mar 16;13:1092602. doi: 10.3389/fonc.2023.1092602 (PMC10061142; doi:10.3389/fonc.2023.1092602)
Supplement: Supplementary file 1 [file Table_1.doc]

**Supplementary Table 1. Trends in Age-Standardized Incidence Rates for Woman’**s Cancers in Changle According to the Joinpoint Analysis in 1988 - 2015

| Site | Trend | | R^2^ |
| --- | --- | --- | --- |
|  | Period | APC(95%CI) |  |
| Breast | 1988-2004 | 5.6*(2.2，9.0) | 0.92 |
|  | 2004-2008 | -7.7(-47.1,61.0） |  |
|  | 2008-2011 | 13.7(-34.8,98.3) |  |
|  | 2011-2015 | -0.3(-13.5,15.0) |  |
| Cervix | 1988-2005 | 18.3*(12.7,24.2) | 0.90 |
|  | 2005-2015 | 1.8(-2.8,6.7) |  |
| Corpus uteri | 1988-2015 | 5.1*(1.8,8.6) | 0.41 |
| Ovary | 1988-2015 | 5.1*(3.0,7.2) | 0.57 |

Abbreviations: APC,annual percent change(%); 95CI, confidence interval.

* The annual percent change is significantly different from 0 (two-side p < 0.05).

**Supplementary Table 2. Trends in Age-Standardized Mortality Rates for Woman’s Cancers in Changle According to the Joinpoint Analysis in 1988 - 2015**

| Site | Trend | | R^2^ |
| --- | --- | --- | --- |
|  | Period | APC(95% CI) |  |
| Breast | 1988-2015 | 0.6 (-0.6, 1.9) | 0.04 |
| Cervix | 1988-2000 | 2.5 (-12.7, 20.4) | 0.86 |
|  | 2000-2007 | 15.5 (-3.7, 38.4) |  |
|  | 2007-2015 | -0.1 (-4.9,5.0) |  |
| Corpus uteri | 1988-2005 | -25.5 (-46.8,4.3) | 0.53 |
|  | 2005-2015 | 47.9* (22.8,78.1) |  |
| Ovary | 1988-2015 | 3.7 (-4.8,12.9) | 0.04 |

Abbreviations: APC,annual percent change; CI, confidence interval.

*The annual percent change is significantly different from 0 (two-side p < 0.05).

**Supplementary Table 3. Trends in Age-Standardized Incidence and Mortality Rates for Woman’s Cancers in Changle**

| Site | Period1 | APC(95%CI) | Period 2 | APC(95%CI) | Period 3 | APC(95%CI) | Period 4 | APC(95%CI) |
| --- | --- | --- | --- | --- | --- | --- | --- | --- |
| Breast |  |  |  |  |  |  |  |  |
| Incidence | 1988-2002 | 5.9*(4.2,7.6) | 2003-2007 | -4.9(13.5,4.6) | 2008-2012 | 13.3*(8.8,18.1) | 2013-2015 | 13.8*(0.6,28.8) |
| Mortality | 1988-2002 | 2.1(-13.2,20.2) | 2003-2007 | -4.9(-14.8,6.1) | 2008-2012 | 10.2(-9.9,34.9) | 2013-2015 | -7.6(-78.4,295.7) |
| Cervix |  |  |  |  |  |  |  |  |
| Incidence | 1988-2002 | 13.1(-28.9,79.9) | 2003-2007 | 4.6*(1.5,7.9) | 2008-2012 | -4.6(-22.0,16.6) | 2013-2015 | -9.6(-20.9,3.4) |
| Mortality | 1988-2002 | 1.3(-19.9,28.2) | 2003-2007 | 19.5(-9.8,58.4) | 2008-2012 | 2.4(-6.8,12.5) | 2013-2015 | -5.5(-31.4,30.2) |
| Corpus uteri |  |  |  |  |  |  |  |  |
| Incidence | 1988-2002 | -9.7*(-9.7,-9.7) | 2003-2007 | -10.0(-69.5,165.7) | 2008-2012 | 5.5(-33.6,67.5) | 2013-2015 | -19.0(-70.1,119.1) |
| Mortality | 1988-2002 | -21.7*(-27.4,-15.5) | 2003-2007 | 3.1(-99.9,137000.1) | 2008-2012 | 51.5*(0.8,127.7) | 2013-2015 | 16.9*(10.4,23.7) |
| Ovary |  |  |  |  |  |  |  |  |
| Incidence | 1988-2002 | 6.5(-3.6),17.7) | 2003-2007 | -4.9(-13.5,4.6) | 2008-2012 | 13.8*(8.8,18.1) | 2013-2015 | 13.8(0.6,28.8) |
| Mortality | 1988-2002 | 2.2(-20.9,32.1) | 2003-2007 | -4.9(-14.8,6.1) | 2008-2012 | 10.2(-9.9,34.9) | 2013-2015 | -7.6(-78.4,295.7) |

Abbreviations: APC,annual percent change
